# Activation of Mu Opioid Receptors Sensitizes Transient Receptor Potential Vanilloid Type 1 (TRPV1) via β-Arrestin-2-Mediated Cross-Talk

**DOI:** 10.1371/journal.pone.0093688

**Published:** 2014-04-02

**Authors:** Matthew P. Rowan, Sonya M. Bierbower, Michael A. Eskander, Kalina Szteyn, Elaine D. Por, Ruben Gomez, Nicholas Veldhuis, Nigel W. Bunnett, Nathaniel A. Jeske

**Affiliations:** 1 Department of Oral and Maxillofacial Surgery, The University of Texas Health Science Center at San Antonio, San Antonio, Texas, United States of America; 2 Department of Pharmacology, The University of Texas Health Science Center at San Antonio, San Antonio, Texas, United States of America; 3 Department of Physiology, The University of Texas Health Science Center at San Antonio, San Antonio, Texas, United States of America; 4 Departments of Pharmacology and Medicine, Monash Institute of Pharmacological Sciences, Parkville, Victoria, Australia; University of South California, United States of America

## Abstract

The transient receptor potential family V1 channel (TRPV1) is activated by multiple stimuli, including capsaicin, acid, endovanilloids, and heat (>42C). Post-translational modifications to TRPV1 result in dynamic changes to the sensitivity of receptor activation. We have previously demonstrated that β-arrestin2 actively participates in a scaffolding mechanism to inhibit TRPV1 phosphorylation, thereby reducing TRPV1 sensitivity. In this study, we evaluated the effect of β-arrestin2 sequestration by G-protein coupled receptors (GPCRs) on thermal and chemical activation of TRPV1. Here we report that activation of mu opioid receptor by either morphine or DAMGO results in β-arrestin2 recruitment to mu opioid receptor in sensory neurons, while activation by herkinorin does not. Furthermore, treatment of sensory neurons with morphine or DAMGO stimulates β-arrestin2 dissociation from TRPV1 and increased sensitivity of the receptor. Conversely, herkinorin treatment has no effect on TRPV1 sensitivity. Additional behavioral studies indicate that GPCR-driven β-arrestin2 sequestration plays an important peripheral role in the development of thermal sensitivity. Taken together, the reported data identify a novel cross-talk mechanism between GPCRs and TRPV1 that may contribute to multiple clinical conditions.

## Introduction

The transient receptor potential V1 channel (TRPV1) is a nonselective ligand-gated ion channel that is activated by a variety of stimuli, including the selective agonist capsaicin (CAP) and temperatures greater than 42°C, to gate calcium influx [1], [2]. TRPV1 has six transmembrane domains with both N-and C-termini located intracellularly, providing multiple targets for post-translational phosphorylation [1]. Phosphorylation of TRPV1 by multiple kinases, including protein kinases A (PKA) [3] and C [4], sensitizes TRPV1 to activation by chemical and thermal agonists resulting in thermal hyperalgesia in behavioral models [5]. Conversely, dephosphorylation of TRPV1 desensitizes the channel [6], providing a dynamic molecular model for manipulating mechanisms thought to precipitate inflammatory hyperalgesia. Recent studies have identified β-arrestins as novel regulators of the function of several TRP channels, establishing a significant role for the arrestin molecule in ionotropic receptor desensitization [7]–[9]. β-arrestin molecules were originally identified as important mediators of metabotropic receptor desensitization, governing internalization of G-protein coupled receptors (GPCRs) following agonist exposure [10]. However, recent reports identify that β-arrestin2 also serves to scaffold phosphodiesterase PDE4D5 within close spatial proximity to TRPV1, thereby reducing PKA phosphorylation and effectively desensitizing the ionotropic receptor [8]. Despite this finding, no one has evaluated β-arrestin2-dependent cross-talk mechanisms between GPCRs and ionotropic receptors such as TRPV1. Multiple GPCRs are co-expressed with TRPV1 in various neuronal populations. In order to accurately evaluate β-arrestin2 cross-talk between a GPCR and TRPV1, we ruled out receptor systems coupled to Gαs and Gαq, which would stimulate kinases that sensitize TRPV1 and confound result interpretation. Therefore, we chose to examine whether the activation of the Gαi-coupled mu opioid receptor (MOPr) alters TRPV1 desensitization by β-arrestin2 scaffolding mechanisms. Indeed, these receptor systems provide an optimal environment for investigation, given that MOPr signals primarily through Gαi proteins to inhibit adenylyl cyclase activity [11], and is co-expressed with TRPV1 in sensory neurons of the dorsal root and trigeminal (TG) ganglia [12]. Furthermore, mice lacking β-arrestin2 display enhanced and prolonged antinociception in response to morphine [13], [14], and reduced tolerance to systemically [13], [15] and peripherally [16] administered morphine. Conversely, wild-type mice and humans that receive chronic morphine treatment develop thermal sensitivity in the periphery, a classic symptom of opioid-induced hyperalgesia [17].

In this study, selective pharmacological agonists of MOPr were employed to dissect potential β-arrestin2 cross-talk mechanisms with TRPV1. Specifically, we used the prototypical MOPr-selective agonists [D-Ala^2^, N-MePhe^4^, Gly-ol^5^]-enkephalin (DAMGO) and morphine, which stimulate receptor desensitization in a β-arrestin2-dependent manner, and herkinorin, a highly selective MOPr agonist that produces full agonist responses but does not recruit β-arrestin2 [18]. Importantly, differential MOPr sequestration of β-arrestin2 following DAMGO, morphine, and herkinorin treatment identify a novel cross-talk mechanism between MOPr and TRPV1 in sensory neurons. Furthermore, this mechanism establishes a role for β-arrestin2 as a contributor to the development of opioid-induced hyperalgesia.

## Materials and Methods

### Materials

Herkinorin was provided by Tom Prisinzano (University of Iowa) and purchased from Abcam (Cambridge, MA). Prostaglandin E2 was from Cayman Chemical (Ann Arbor, MI). All tissue culture reagents and culture media were from Invitrogen (Grand Island, NY) unless otherwise indicated. Other drugs and chemicals were from Sigma Aldrich (St. Louis, MO) unless otherwise indicated.

### Animals

All procedures using animals were approved by the Institutional Animal Care and Use Committee of The University of Texas Health Science Center at San Antonio and were conducted in accordance with policies for the ethical treatment of animals established by the National Institutes of Health and International Association for the Study of Pain. Male C57BL6 mice (22–25 g), TRPV1 knockout mice (22–25 g), and male Sprague-Dawley rats (175–200 g) used in these studies were from Charles River (Wilmington, MA).

### Behavior

All injections were given intraplantarly in 50 μl (rat) or 10 μl (mouse) volumes via a 28-gauge needle inserted through the lateral footpad just under the skin to minimize tissue damage. Drug stocks were dissolved in PBS, or PBS with 2% Tween20 (for experiments with DMSO). Paw withdrawal latency to a thermal stimulus was measured with a plantar test apparatus (IITC, Woodland Hills, CA) as described [19]. Nocifensive behavior in response to CAP (Tocris Bioscience, Minneapolis, MN; 0.5 μg and 0.1 μg for rats and mice, respectively) was defined as hindpaw lifting, flinching, or licking and was quantified for 5 min as described [20], [21].

### Primary TG Neuron Culture

Rat TG neurons were chosen as a model over dorsal root ganglion neurons due to their similarity in MOPr/TRPV1 co-expression [12], [22]–[25] and to reduce the number of animals required for this study, and were cultured as described [26], [27]. Briefly, rats were sacrificed by decapitation, and freshly dissociated TG neurons were digested with collagenase and trypsin. Cells were centrifuged, enzymes were aspirated, and the cell pellet was re-suspended in Dulbecco’s modified Eagle’s medium supplemented with 10% fetal bovine serum, 100 ng/ml nerve growth factor (Harlan Laboratories, Indianapolis, IN), 1% penicillin/streptomycin and 1% glutamine, then placed on poly-D-lysine coated 10 cm plates (BD Biosciences, San Jose, CA; co-immunoprecipitation), or poly-D-lysine- and laminin- coated coverslips (BD Biosciences, San Jose, CA; electrophysiology, calcium imaging) or glass bottom plates (MatTek, Ashland, MA; TIRF-FRET). Cultures were maintained at 37°C, 5% CO_2_ for 5–7 d for co-immunoprecipitation experiments and 1–2 d for calcium imaging, electrophysiology, and total internal reflective fluorescence-Forster resonance energy transfer (TIRF-FRET) experiments. TGs were transfected with β-arrestin2 siRNA as described [8].

### Nucleofection

For experiments with coverslips or glass bottom plates, 3 μg cDNAs for MOPr-GFP (Dr. Marc Caron, Duke), MOPr-YFP or β-arrestin2-CFP (Dr. Eamonn Kelly, Bristol) were nucleofected (Lonza, Allendale, NJ) prior to plating on the day of culture for primary rat neurons. Transfection efficiency was approximately twenty percent (data not shown) as reported elsewhere [28], [29].

### Calcium Imaging

Changes in intracellular calcium levels were measured using Fura-2AM (Invitrogen) as described [8]. Briefly, cultured TG neurons on poly-D-lysine-laminin-coated coverslips were incubated with Fura-2AM (2 μM) for 30 min at 37°C in the presence of 0.05% Pluronic (EMD Millipore, Philadelphia, PA) in a standard extracellular solution (SES) containing (in mM): 140 NaCl, 5 KCl, 2 CaCl_2_, 1 MgCl_2_, 10 D-glucose, pH 7.4. Nucleofected cells were identified by the presence of GFP. Intracellular calcium accumulation was measured following TRPV1 activation with CAP (50 nM). Fluorescence was detected with a Nikon Eclipse Ti-U microscope fitted with a X20/0.8 Na Fluor objective, and images from 340 and 380 nm excitation wavelengths were collected and analyzed with MetaFluor Software (MetaMorph). The net change in calcium (ΔF340/380) was calculated by subtracting the basal F340/380 ratio from the peak F340/380 achieved during stimulation.

### Electrophysiology (CAP Inhibition)

All *I*_CAP_ recordings from the somata of cultured TGs (15–60 pF) were made in the whole cell patch configuration with a holding potential (V_h_) of −60 mV to represent physiological resting membrane potential. Data were acquired and analyzed with an Axopatch200B amplifier and pCLAMP 10 software (Molecular Devices, Union City, CA) with recordings filtered at 0.5 kHz and sampled at 2 kHz. Borosilicate pipettes (Sutter Instruments, Novato, CA) were polished to resistances of 5–10 MΩ in pipette solution. Access resistance was compensated (40–80%) when appropriate. SES was the same as for calcium imaging experiments supplemented with 2 mM Ca^2+^. Pipette solution contained SES supplemented with 0.2 mM Na-GTP, 2.5 mM Mg-ATP, and 1 mM CaCl_2_ (pH 7.3) as described [30]. CAP (100 nM) was locally applied using a computer-controlled application system (MicroData Instrument, South Plainfield, NJ).

### Electrophysiology (Calcium Channels)

Opioid-mediated inhibition of N-type voltage-gated calcium channels was measured as described [31]. Briefly, patch clamp capillaries (2–4 MΩ) were pulled from borosilicate glass (World Precision Instruments, Sarasota, FL) with a micropipette puller (Narishige, Japan). Currents were measured in whole-cell configuration with an EPC-10 amplifier (HEKA, Germany) and analyzed with Patch Master software (HEKA). Giga seal and whole-cell configuration were established in SES containing (in mM): 145 NaCl, 5 KCl, 2 MgCl_2_, 2CaCl_2_, 10 HEPES, and 10 glucose (pH 7.4), then changed to extracellular solution containing (in mM): 140 NMDG-Cl, 2 MgCl_2_, 3 BaCl_2_, 10 HEPES, and 10 glucose. Pipette solution contained (in mM): 120 CsCl, 1 MgCl_2_, 10 EGTA, 10 HEPES, 4 Mg-ATP, 0.3 Na-GTP (pH 7.2). The voltage-gated Ca^2+^ currents, carried by Ba^2+^, were activated by pulses from −70 to 25 mV (150 ms, 5 mV steps, 5 s intervals) from a holding potential of −70 mV. DAMGO (1 μM) and herkinorin (10 μM) were applied via bath application and only cells that showed reversible effects of drug treatment were included in analysis. The identity of the currents was verified with application of the N-type calcium channel inhibitor α-conotoxin (Alomone, Israel).

### TIRF-FRET

Fluorescence images were obtained using TIRF microscopy paired with FRET energy transfer to examine protein-protein interactions near the plasma membrane of TG neurons nucleofected with MOPr-YFP and β-arrestin2-CFP as described [8], [32]. Fluorescent emission from CFP- or YFP-tagged proteins was collected at room temperature using an inverted Eclipse Ti Microscope with through-the-lens TIRF imaging (Nikon, Melville, NY) fitted with a Plan Apo TIRF 60x/1.45NA oil immersion high-resolution objective and a vibration isolation system (Technical Manufacturing, Peabody, MA) to minimize drift and noise. Prior to imaging, the medium was changed from serum free culture medium to SES containing morphine (1 μM), DAMGO (1 μM), herkinorin (10 μM), or vehicle (0.1% DMSO). Cells were first examined using the mercury lamp and standard CFP or YFP filter cubes to locate a cell suitable for imaging. Under TIRF illumination, the focal plane was adjusted if necessary immediately before each image acquisition to obtain a sharp TIRF image. TIRF images were collected (300 ms exposure time) using 442 and 514 nm laser lines before and after photobleaching of the YFP fluorophores. Images were not binned nor filtered, with pixel size corresponding to a square of 122×122 nm.

### Co-immunoprecipitation and Western Blot

TG neuron cultures were pretreated as indicated, harvested by scraping, and homogenized. Plasma membrane homogenates were isolated and total protein was quantified using the Bradford assay. Equal amounts of protein from each treatment condition were immunoprecipitated and analyzed by Western blot as described previously [8] using antibodies (Santa Cruz Biotechnology, Dallas, TX) to TRPV1 (R130) or β-arrestin2 (H9).

### Data Analyses and Statistics

Results were analyzed using GraphPad Prism 5.0 (La Jolla, CA). Data are presented as mean ± SEM, with *n* referring to the number of analyzed cells, plates, or animals per group. Significance was assessed by one-way or two-way analysis of variance (ANOVA) with Bonferroni post hoc correction.

## Results

### Pretreatment with Morphine or DAMGO, not Herkinorin, Increases MOPr Association with β-arrestin2 in Primary Sensory Neurons

**Figure 1:**
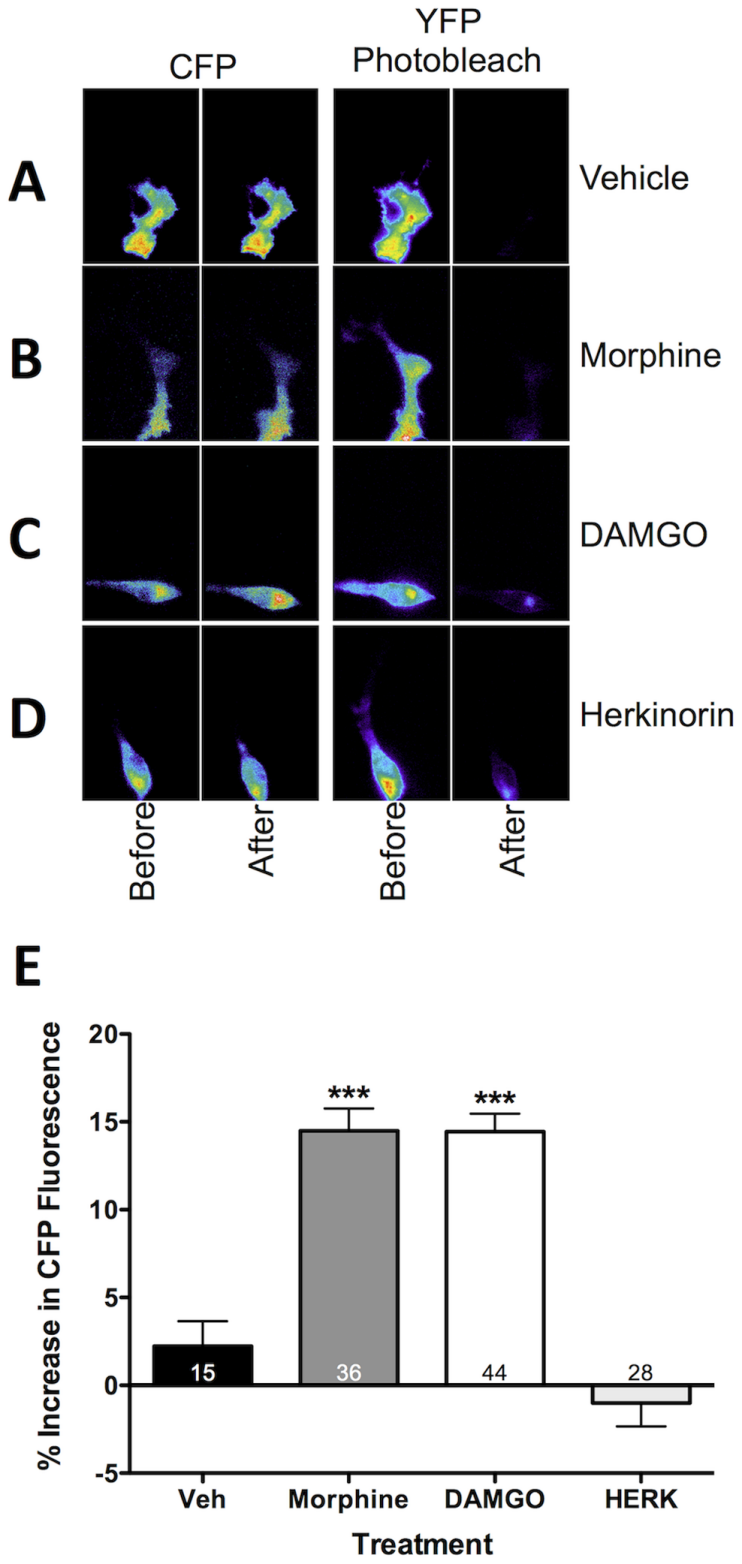
Morphine and DAMGO, not herkinorin, recruit β-arrestin2 to MOPr in primary sensory neurons. TG neurons from rats were nucleofected with MOPr-YFP and β-arrestin2-CFP and pretreated with morphine (1 μM), DAMGO (1 μM), herkinorin (10 μM), or vehicle (0.1% DMSO) for 15 min. Cells were rinsed and the interaction of MOPr and β-arrestin2 was measured by TIRF-FRET microscopy. **A–D)** Images of TGs under TIRF illumination expressing MOPr-YFP and β-arrestin2-CFP under 442- and 514-nm laser lines. Pseudocolor images of CFP (left) and YFP (right) emissions are shown before and after YFP photobleaching. **E)** Mean ± SEM percentage increase in CFP emission after YFP photobleaching for the number of cells indicated on each bar. ***, p<0.001 by one-way ANOVA.

Morphine is much less effective than other MOPr agonists at recruiting β-arrestin2 to MOPr in transfected cell models [33], [34]. However, morphine recruits β-arrestin2 to MOPr when G-protein-coupled receptor kinase 2 (GRK2) is overexpressed in heterologous cell models [35]. Furthermore, morphine recruits β-arrestin2 to MOPr in striatal neurons [36], and may promote increased recruitment of β-arrestin2 to MOPr and subsequent internalization following chronic treatment of neurons [37]. To evaluate the extent of morphine- and DAMGO-mediated recruitment of β-arrestin2 to MOPr in sensory neurons, cultured TG neurons were nucleofected with MOPr-YFP and β-arrestin2-CFP cDNAs and observed under TIRF-FRET microscopy following pretreatment with morphine (1 μM, 15 min), DAMGO (1 μM, 15 min), herkinorin (10 μM, 15 min), or vehicle (0.1% DMSO). Notably, these concentrations are in agreement with previous studies [18], and follow non-acute treatment timelines more similar to chronic agonist exposure. TIRF-FRET was used here since the general non-specificity of commercially available antibodies for MOPr prevent co-immunoprecipitation. Sensory neurons treated with vehicle or herkinorin revealed no significant difference in CFP emission (Figs. 1A, 1D, and 1E). However, neurons treated with morphine or DAMGO displayed significant increases in CFP emission (14.49% and 14.45%, respectively; Figs. 1B, 1C, and 1E), indicating increased association between MOPr and β-arrestin2 at the plasma membrane. For comparison, TG neurons nucleofected with a positive control, plasma membrane-targeted CFP-YFP tandem protein, Rho-pYC, displayed a 25±6% increase in CFP emission over baseline, consistent with previous results in non-neuronal cells [8]. As a control, all neurons tested had YFP photobleach values above 80% (data not shown). For the first time, TIRF-FRET microscopic analyses indicate that morphine and DAMGO, not herkinorin, are capable of recruiting β-arrestin2 to MOPr in sensory neurons.

### Pretreatment with Morphine or DAMGO, not Herkinorin, Decreases TRPV1 Association with β-arrestin2

**Figure 2:**
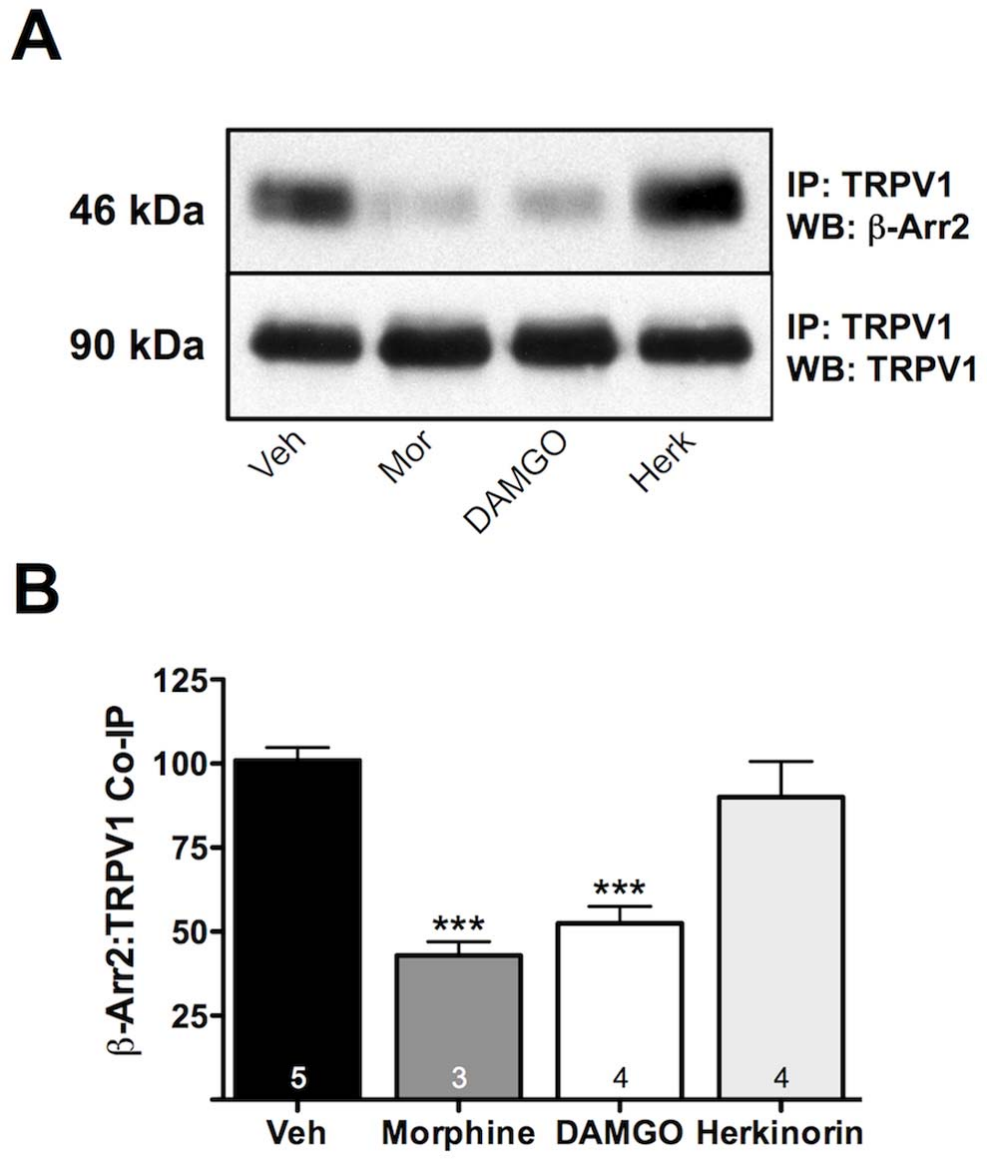
Treatment of primary sensory neurons with morphine or DAMGO, not herkinorin, decreases TRPV1 association with β-arrestin2. TG neurons from rats were pretreated with morphine (1 μM), DAMGO (1 μM), herkinorin (10 μM), or vehicle (0.1% DMSO) for 15 min. Cells were rinsed and association of TRPV1 and β-arrestin2 was assessed via co-immunoprecipitation and Western blot (WB) analysis. **A)** Representative blot following immunoprecipitation (IP) for TRPV1 and probe (WB) for β-arrestin2 (β-Arr2) or TRPV1. **B)** Mean ± SEM from 3–5 independent experiments per treatment condition. *** p<0.001 by one-way ANOVA.

Since β-arrestin2 natively associates with TRPV1 [8], we examined whether agonist activation of MOPr would recruit β-arrestin2 away from TRPV1. Therefore, we pretreated cultured sensory neurons with morphine (1 μM, 15 min), DAMGO (1 μM, 15 min), herkinorin (10 μM, 15 min), or vehicle (0.1% DMSO) and quantified TRPV1 association with β-arrestin2 by co-immunoprecipitation. In TG cultures treated with vehicle, β-arrestin2 and TRPV1 associated strongly, consistent with earlier findings [8]. However, TRPV1 association with β-arrestin2 decreased following pretreatment with morphine or DAMGO (Figs. 2A and 2B). Herkinorin does not recruit β-arrestin2 to MOPr in sensory neurons (Fig. 1) or in heterologous cell models [18] and, as expected, did not affect TRPV1 association with β-arrestin2 (Figs. 2A and 2B). Together, these data demonstrate that either morphine or DAMGO attenuates β-arrestin2 association with TRPV1.

### Pretreatment with Morphine or DAMGO, not Herkinorin, Increases TRPV1-response in a β-arrestin2- and PKA-dependent Manner

**Figure 3:**
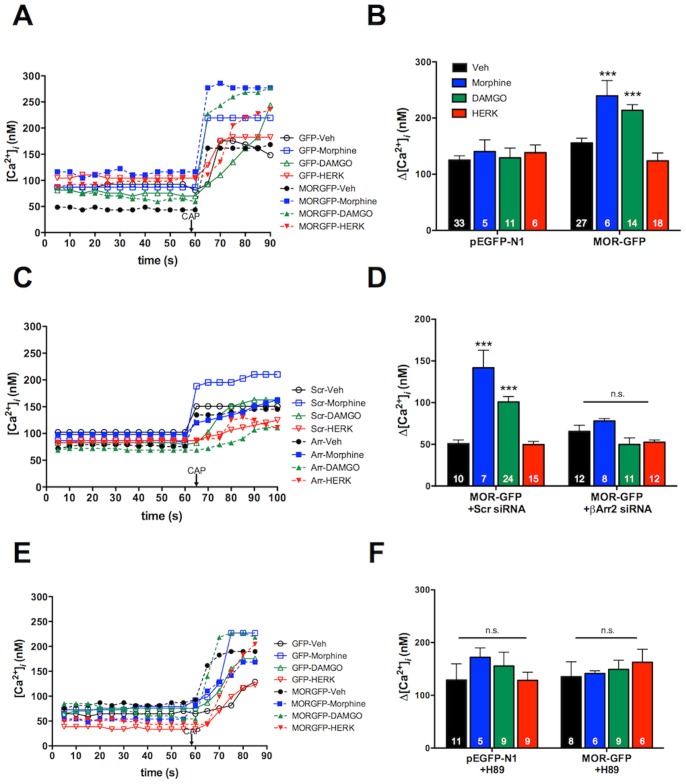
Pretreatment with morphine or DAMGO increases capsaicin responses in primary sensory neurons, and requires β-arrestin2 and PKA activation. **A, B)** TG neurons from rats were nucleofected with MOPr-GFP or empty vector (GFP), and treated with morphine (1 μM), DAMGO (1 μM), herkinorin (10 μM), or vehicle (0.1% DMSO) for 15 min. Real-time calcium responses from individual GFP-positive cells were measured before and after exposure to capsaicin (CAP, 50 nM) and the net change in intracellular calcium accumulation (Δ[Ca^2+^]_i_) was determined. Representative traces (**A**) and the mean ± SEM (**B**) of the difference in pre- and post-capsaicin (CAP) response for the number of cells indicated at the bottom of each bar (B). *,** p<0.05, 0.01 by two-way ANOVA. **C, D)** TG neurons from rats were nucleofected with MOPr-GFP. 24 h later, cells were transfected with siRNA targeting β-arrestin2 (+βArr2 siRNA) or scrambled siRNA (+Scr siRNA) for 18 h. Cells were rinsed with serum free media prior to treatment with morphine, DAMGO, herkinorin, and capsaicin as in **A** and **B**. **,*** p<0.01, 0.001 by two-way ANOVA. **E, F)** TG neurons from rats were nucleofected with MOPr-GFP, and pretreated with the PKA inhibitor, H89 (20 μM), 5 min before treatment with morphine, DAMGO, herkinorin, and capsaicin as in **A** and **B**.

**Figure 4:**
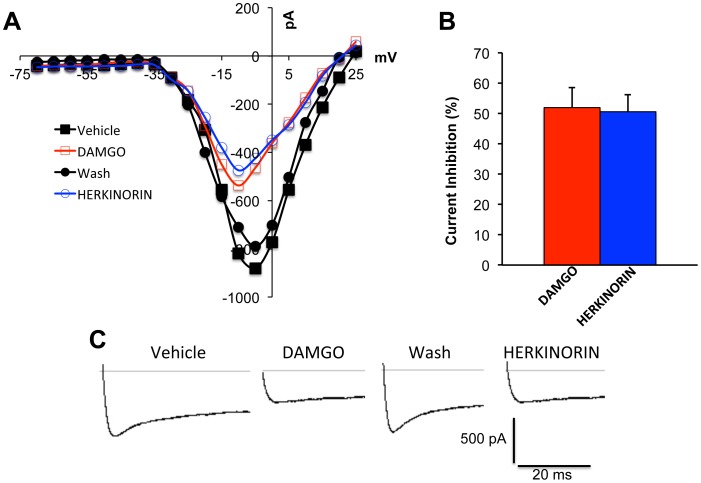
DAMGO and herkinorin inhibit N-type voltage-gated calcium channels in rat sensory neurons. N-type voltage-gated calcium currents were recorded from rat TG neurons 24–48 h after isolation. Maximal current amplitudes were observed at −10 mV. Control currents were recorded first in SES with 0.1% DMSO (Vehicle). Following bath application of DAMGO (1 μM, 5 min) currents were again recorded. Cells that responded to DAMGO were washed with SES (5 min) and currents were recorded again prior to bath application of herkinorin (10 μM, 5 min) and final current measurement. Representative single cell current-voltage curves (**A**), traces (**C**), and the extent of current inhibition (expressed as % inhibition) mean ± SEM of 10 cells (**B**).

**Figure 5:**
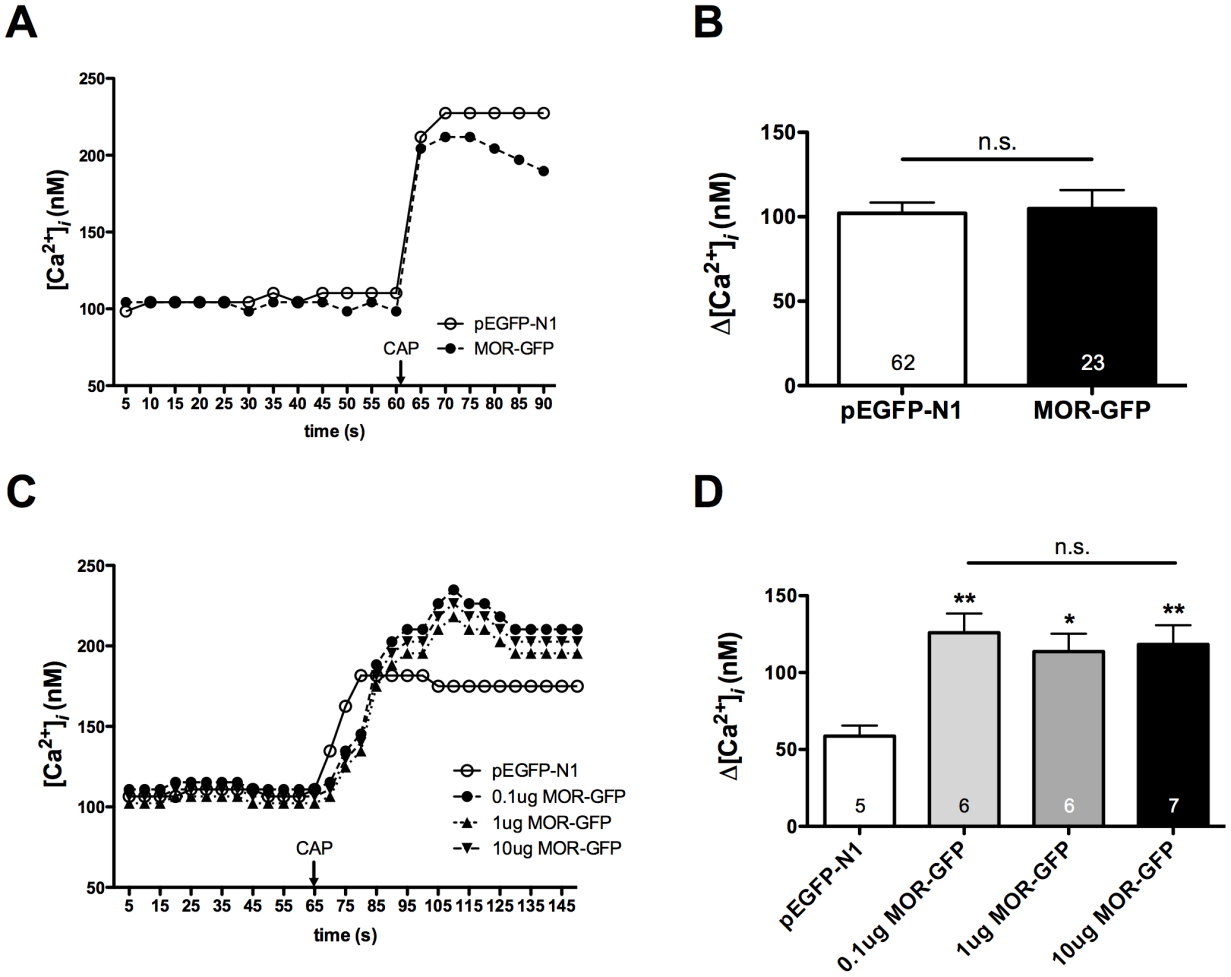
Overexpression of MOPr-GFP does not affect CAP response. **A, B)** TG neurons from rats were nucleofected with MOPr-GFP (1 μg) or empty vector (pEGFP-N1, 1 μg) and the net change in intracellular calcium accumulation (Δ[Ca^2+^]_i_) was determined in GFP-positive cells following exposure to capsaicin (CAP, 100 nM). Representative traces (**A**) and the mean ± SEM (**B**) of the difference in pre- and post-CAP response for the number of cells indicated at the bottom of each bar. n.s. not significant. **C, D)** TG neurons from rats were nucleofected with empty vector (pEGFP-N1, 1 μg) or varying amounts of MOPr-GFP cDNA (0.1, 1, or 10 μg), and treated with DAMGO (1 μM) for 15 min. Real-time calcium responses from GFP-positive cells were measured before and after exposure to CAP (50 nM). Representative traces (**C**) and the mean ± SEM (**D**) of the difference in pre- and post-CAP response for the number of cells indicated at the bottom of each bar. n.s. not significant. *, ** p<0.05, 0.01 vs pEGFP-N1 by one-way ANOVA.

Previous studies have established that β-arrestin2, not β-arrestin1, association with TRPV1 reduces PKA phosphorylation and sensitization of TRPV1 [8]. Therefore, we evaluated the potential for functional cross-talk between MOPr and TRPV1 in primary sensory neurons by measuring TRPV1-mediated calcium accumulation. Importantly, sensory neurons were nucleofected with GFP-tagged MOPr cDNA to identify MOPr-positive neurons, since other measures for identifying MOPr-expression would compromise TRPV1 responses [38]. Hence, cultured TG sensory neurons were nucleofected with MOPr-GFP cDNA (MOPr-GFP) or empty pEGFP-N1 vector (GFP), and TRPV1 responses to CAP (50 nM) in GFP-positive neurons were measured by real-time calcium imaging following pretreatment with morphine (1 μM, 15 min), DAMGO (1 μM, 15 min), herkinorin (10 μM, 15 min), or vehicle (0.1% DMSO). Pretreatment with either morphine or DAMGO resulted in significantly greater CAP responses compared to neurons pre-treated with vehicle (Figs. 3A and 3B). However, pretreatment with herkinorin had no effect on CAP response (Figs. 3A and 3B). DAMGO (1 μM) and herkinorin (10 μM) display equal efficacy in heterologous expression systems [18], [39], but we verified that DAMGO and herkinorin display equal efficacy in primary cells by measuring the acute inhibition of N-type voltage-gated sodium channels [31]. Brief exposure to DAMGO (1 μM, 5 min) or herkinorin (10 μM, 5 min) inhibited N-type voltage-gated sodium channels by approximately 50% (Fig. 4), demonstrating that DAMGO and herkinorin are equally efficacious in primary cells at the concentrations chosen for the *in vitro* studies. Control experiments with empty vector (pEGFP-N1) and MOPr-GFP verified that nucleofection did not affect basal CAP response in the absence of MOPr agonist pretreatment (Figs. 5A and 5B). Importantly, ratiometric transfection control experiments showed that the enhancement of CAP response following pretreatment with DAMGO (1 μM, 15 min) was not due to overexpression of MOPr (Figs. 5C and 5D).

Given the differential ability of morphine and DAMGO versus herkinorin to recruit β-arrestin2 in sensory neurons (Fig. 1) and heterologous expression systems [18], we repeated the experiment following knock-down of β-arrestin2 with previously vetted, FITC-labeled β-arrestin2 siRNA that results in a 65% decrease in the expression of β-arrestin2 in TG neurons but no change in TRPV1 surface expression [8]. Morphine and DAMGO sensitization of the TRPV1 response in neurons was lost following β-arrestin2 knock-down, but unaffected in cells transfected with mismatch siRNA (Figs. 3C and 3D). We have previously shown that β-arrestin2 functions as a scaffold for phosphodiesterase PDE4D5 to inhibit PKA-dependent phosphorylation and sensitization of TRPV1 [8]. To examine the role of PKA in our *in vitro* model, TG neurons were pretreated with the PKA inhibitor, H89 (20 μM, 5 min). H89 blocked morphine- and DAMGO-mediated increases in the CAP response but had no effect on basal CAP response in vehicle-treated cells (Figs. 3E and 3F).

### Pretreatment with Morphine or DAMGO Increases TRPV1 Activity in a β-arrestin2-dependent manner

**Figure 6:**
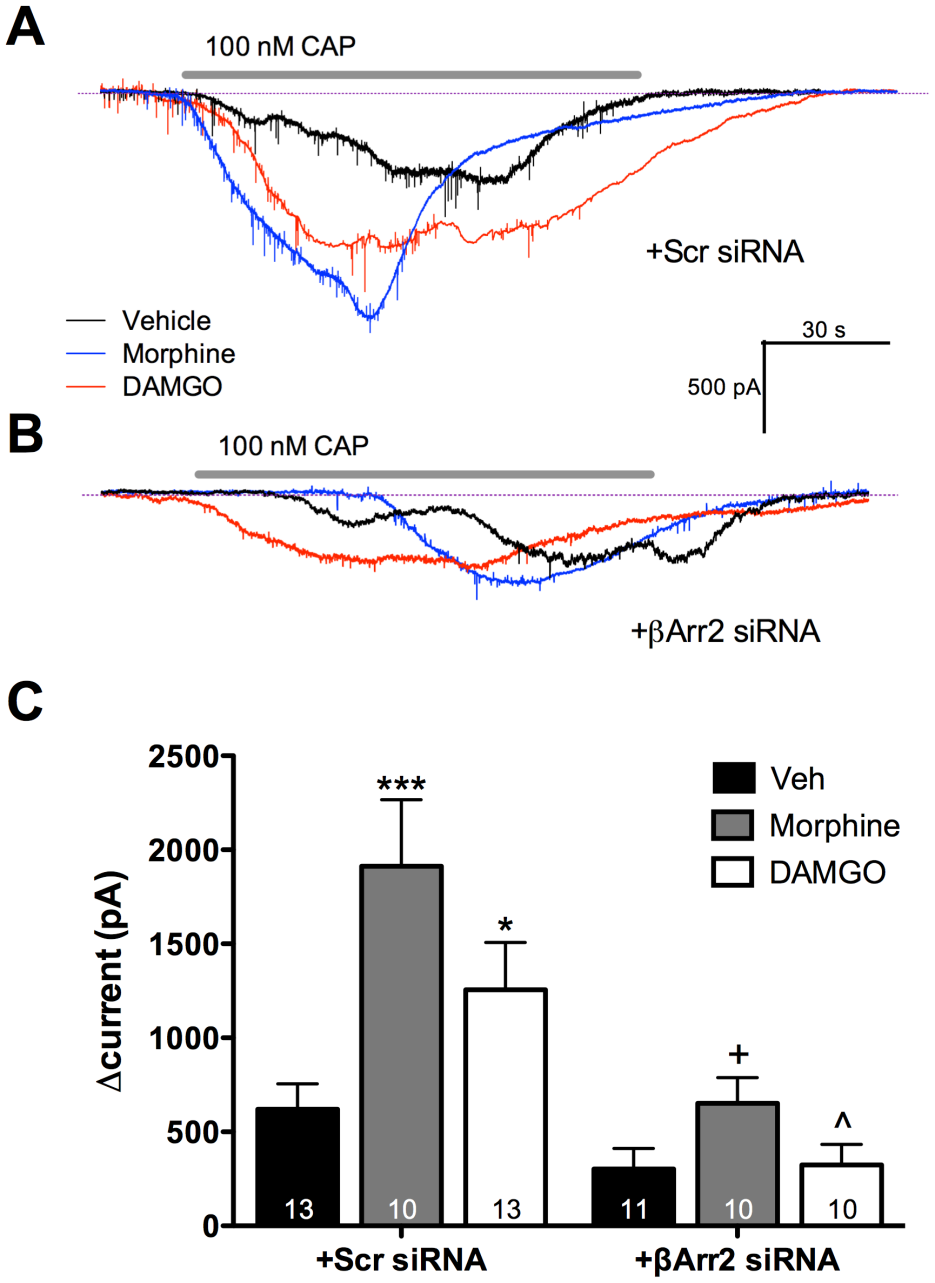
Pretreatment with morphine or DAMGO increases capsaicin-induced currents in primary sensory neurons and requires β-arrestin2. **A)** TG neurons from rats were nucleofected with MOPr-GFP and transfected with scrambled siRNA (+Scr siRNA). Neurons were then treated with morphine (1 μM), DAMGO (1 μM), or vehicle for 15 min. Cells were then patched, and after baseline recordings were exposed to capsaicin (CAP, 100 nM, 1 min). Data shown are representative traces from one cell for each treatment condition. **B)** TG neurons from rats were nucleofected with MOPr-GFP and transfected with siRNA targeting β-arrestin2 (+β-Arr2 siRNA) for 18 h. Cells were rinsed with serum free media, pretreated with morphine or DAMGO, and exposed to CAP as in **A**. Data shown are representative traces from one cell for each treatment condition. The amperage/time legend applies to both panels A and B. **C)** The maximum change in current from baseline (Δcurrent) was calculated for each cell. Bars represent the mean ± SEM for the number of neurons indicated at the bottom of each bar. *,*** p<0.05, 0.001 vs Veh/Scr siRNA; +, p<0.001 vs Morphine/Scr siRNA; ∧, p<0.01 vs DAMGO/Scr siRNA by two-way ANOVA.

To verify that calcium imaging results were due to changes in TRPV1 activity, cultured TG neurons were nucleofected with MOPr-GFP cDNA to visualize MOPr-positive neurons, and cells were pre-treated with morphine (1 μM, 15 min), DAMGO (1 μM, 15 min), or vehicle (0.1% DMSO) as before. Patch clamp recordings were conducted to assess CAP-induced (100 nM) activation of TRPV1 currents. Consistent with calcium imaging experiments, pre-treatment with morphine or DAMGO significantly enhanced CAP currents (Figs. 6A and 6C). Importantly, β-arrestin2 knockdown blocked morphine- and DAMGO-induced increases in TRPV1 activity (Figs. 6B and 6C). Control experiments verified that nucleofection with MOPr-GFP did not affect basal CAP response (data not shown).

### Peripheral Administration of the MOPr Agonists Morphine, DAMGO, or Herkinorin Blocks Thermal Allodynia *in vivo*

**Figure 7:**
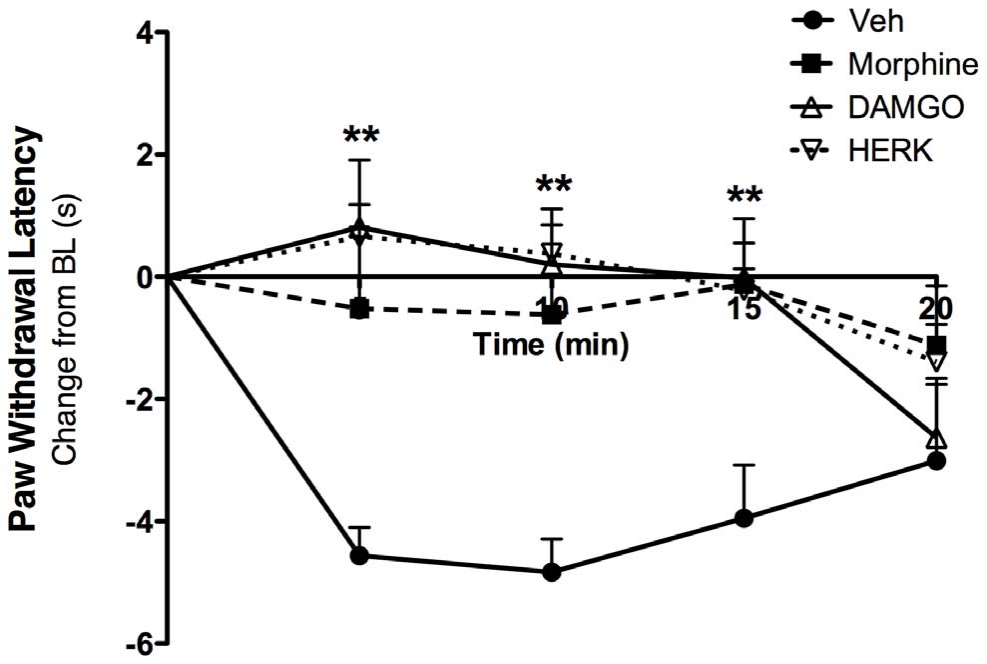
Morphine, DAMGO, and herkinorin inhibit thermal allodynia following peripheral administration. Separate groups of naïve rats received intraplantar bradykinin (25 μg) 15 minutes before co-injection of prostaglandin E2 (300 ng) and morphine (10 μg), DAMGO (2 μg), herkinorin (10 μg) or vehicle. Paw withdrawal latency was measured in duplicate every 5 min for 20 min. Responses were normalized to the pre-injection baseline response for each individual animal, and expressed as the mean change from baseline for 6 rats per group. **, p<0.01 vs Veh by two-way ANOVA.

Under inflammatory conditions, peripherally administered mu, delta, and kappa opioids are highly effective at reducing allodynia following acute injection [40]. To validate the doses chosen for chronic studies, we evaluated whether acute, locally administered morphine, DAMGO, and herkinorin can equally inhibit thermal allodynia employing a model we have used previously [41]. Although previous studies have established that all three agonists fully activate MOPr in *in vitro* models of receptor agonism [18], [33], [36], this evaluation is important to the behavioral validation of MOPr activation with agonists that differentially recruit β-arrestin2 to the receptor. Separate groups of naïve rats received an intraplantar injection of bradykinin (25 μg) to induce acute inflammation [40], followed by an intraplantar injection of the inflammatory mediator prostaglandin E2 (PGE2, 300 ng) with morphine (10 μg), DAMGO (2 μg), herkinorin (10 μg), or vehicle (0.1% DMSO). Paw withdrawal latency to a thermal stimulus was evaluated using the Hargreaves method [19] every 5 min for 20 min following PGE2 injection. As shown in Fig. 7, all three MOPr agonists acutely blocked PGE2-induced thermal allodynia at the doses chosen for this study. Furthermore, the responses seen at the *in vivo* doses chosen for this study are in agreement with what has been shown previously by other groups [42], [43].

### Chronic Peripheral Administration of Morphine or DAMGO, not Herkinorin, Induces OIH

**Figure 8:**
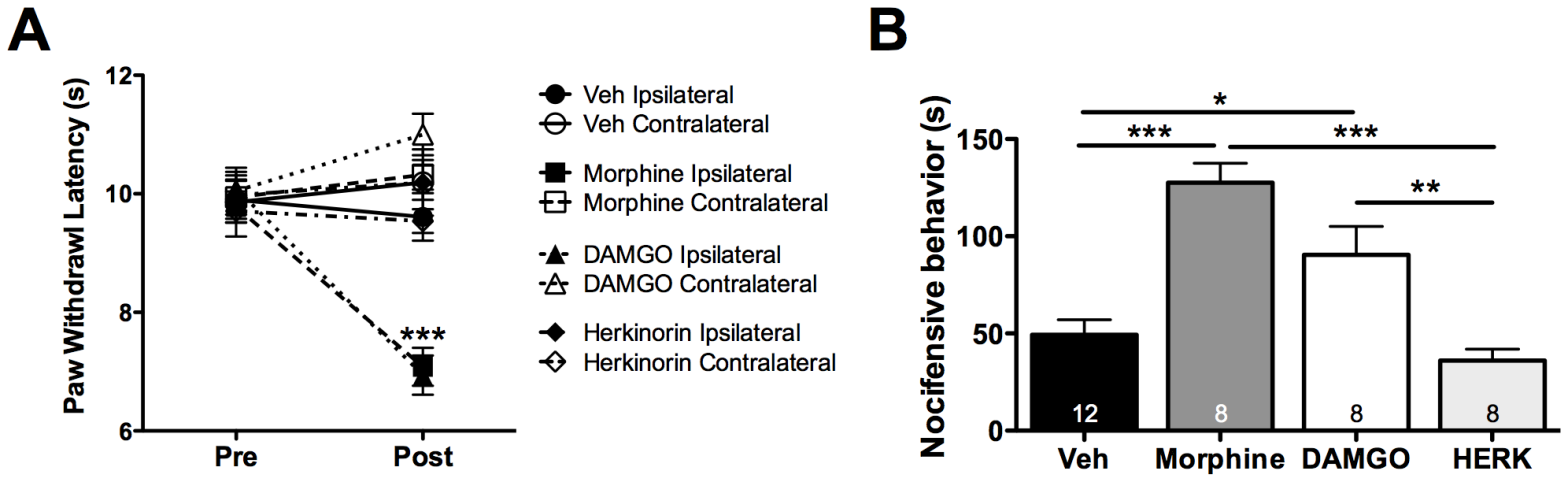
Chronic peripheral administration of morphine or DAMGO, not herkinorin, produces OIH and enhanced TRPV1 responsiveness in rats. **A)** Following baseline thermal testing (Pre), separate groups of rats received intraplantar injections once daily for 5 days of vehicle (Veh, 2% DMSO, 2% Tween20, PBS), morphine (10 μg), DAMGO (2 μg), or herkinorin (HERK, 10 μg). On day 6 (Post), animals were re-tested for thermal sensitivity at least 24 h after the last injection. *** p<0.001 vs upper six groups by two-way ANOVA. n = 8–12 rats/group. **B)** Following thermal testing on day 6, rats received intraplantar capsaicin (0.5 μg) and nocifensive behavior was quantified for 5 min. *, **, *** p<0.05, 0.01, 0.001 by one-way ANOVA. n = 8–12 rats/group.

Chronic administration of MOPr agonists leads to the development of opioid-induced hyperalgesia (OIH) [44], [45], which can be seen as a reduction in nociceptive thresholds in the absence of an inflammatory insult [17], [46]. To determine whether β-arrestin2 cross-talk between MOPr and TRPV1 yields significant behavioral effects, we employed an *in vivo* model of OIH using chronic daily local (intraplantar) injections for 5 days with morphine (10 μg) or DAMGO (2 μg). Although previous evaluations of OIH mechanisms have employed systemic administration of MOPr agonists such as morphine [44], [45], we chose to administer local injections in this study to accurately quantify behavioral changes at the level of the primary sensory neuron. Following daily opioid injection, rats displayed an increased thermal sensitivity, seen as a reduction in thermal paw withdrawal latency in the ipsilateral hindpaw (Fig. 8A), a hallmark symptom of OIH [44]. No changes were observed in the contralateral hindpaw, indicating that the doses chosen and mechanisms involved were restricted to primary sensory neurons. Notably, chronic daily intraplantar injections for 5 days with herkinorin (10 μg) did not produce OIH (Fig. 8A), although, at the selected doses, herkinorin was as effective as morphine and DAMGO at inhibiting acute thermal allodynia (Fig. 7).

**Figure 9:**
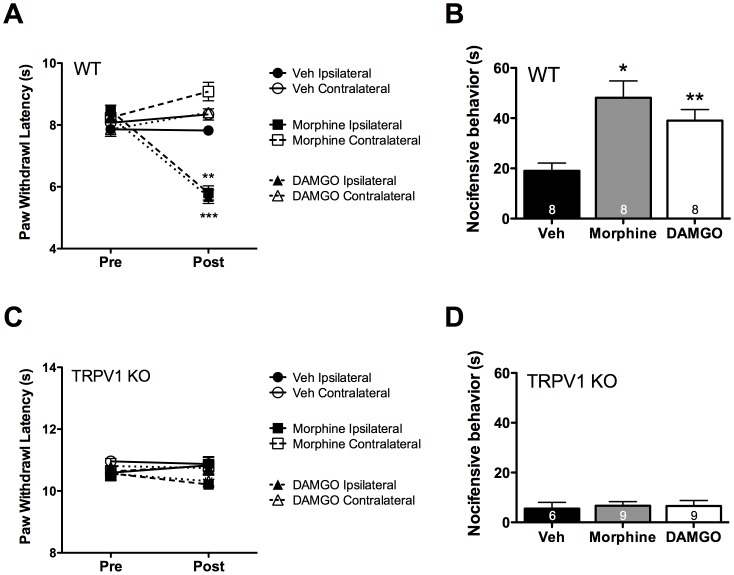
Morphine- and DAMGO-stimulated OIH require TRPV1. **A)** Following baseline thermal testing (Pre), separate groups of wild-type (WT) C57BL6 mice received intraplantar injections once daily for 5 days with vehicle (Veh, PBS), morphine (6 μg), or DAMGO (1 μg). On day 6 (Post), animals were retested for thermal sensitivity at least 24 h after the last injection. **, *** p<0.01, 0.001 vs upper four groups by two-way ANOVA. n = 8 mice/group. **B)** Following thermal testing on day 6, mice were injected with capsaicin (0.1 μg) and nocifensive behavior was quantified for 5 min. *, ** p<0.05, 0.01 vs Veh by one-way ANOVA. n = 8 mice/group. **C, D)** Experiments were performed as in panels **A** and **B**, with TRPV1 knockout (KO) mice, n = 6–9 mice/group.

TRPV1 has been shown to be involved in OIH following systemic morphine administration [45]. Therefore following chronic MOPr agonist administration, rats were given an intraplantar injection of CAP (0.5 μg) and nocifensive behavior (paw lifting, flinching, licking) was quantified for 5 min. Rats chronically injected with morphine (10 μg, 5 days) or DAMGO (2 μg, 5 days), but not herkinorin (10 μg, 5 days), displayed enhanced sensitivity to capsaicin (Fig. 8B). Similarly, mice chronically injected (intraplantarly) with morphine (6 μg, 5 days) or DAMGO (1 μg, 5 days) also developed thermal allodynia and displayed increased sensitivity to capsaicin (CAP, 0.1 μg, Figs. 9A and 9B). However, TRPV1 knock-out mice failed to develop symptoms of OIH (Figs. 9C and 9D) following chronic peripheral administration of MOPr agonists, consistent with previous reports indicating the importance of TRPV1 to OIH in a systemic model [45].

## Discussion

Previous studies have evaluated cross-talk mechanisms involving opioid receptors with other metabotropic GPCR systems. However, to our knowledge this is the first study to identify a β-arrestin2-dependent cross-talk mechanism between MOPr and the ionotropic receptor TRPV1. Using an *in vitro* culture system of primary sensory neurons, we demonstrate, for the first time, that activation of MOPr by DAMGO or morphine leads to recruitment of β-arrestin2 to MOPr, away from TRPV1. Additionally, we demonstrate that the recruitment of β-arrestin2 away from TRPV1 results in sensitization of TRPV1 responses through a β-arrestin2- and PKA-dependent manner. We also show that the MOPr-selective agonist, herkinorin, neither recruits β-arrestin2 to MOPr nor sensitizes TRPV1 responses in sensory neurons. Furthermore, we identify physiologically significant cross-talk between MOPr and TRPV1, as β-arrestin2 recruitment to MOPr sensitizes TRPV1 and contributes to thermal hypersensitivity associated with OIH.

Studies in heterologous expression systems have demonstrated dichotomous effects following MOPr activation by morphine. Although morphine has high efficacy for G-protein-mediated inhibition of adenylyl cyclase and activation of downstream kinases, morphine has low efficacy for phosphorylation of MOPr at Ser375, recruitment of β-arrestin2 to MOPr, or internalization of MOPr unless GRK2 and β-arrestin2 are overexpressed [34], [35]. In contrast, studies of intact animals and neuronal cultures indicate that morphine can signal to β-arrestin2 in native cells [36], [47]. This latter observation is not surprising given that endogenous β-arrestin2 is required for the development of morphine tolerance [13], [48], and mice lacking β-arrestin2 demonstrate increased sensitivity to the antinociceptive effects of morphine [14]. These discrepancies between heterologous expression models and native neurons may be due to the relatively abundant expression of GRK2 in neurons compared to heterologous cell systems [36], [47], and may explain why we observe dramatic changes in MOPr association with β-arrestin2 in cultured sensory neurons that are known to express functional GRK2 [49]. Importantly, our results using TIRF-FRET microscopy in cultured sensory neurons help to define the nature of MOPr interactions with β-arrestin2 at the plasma membrane.

Agonists of MOPr used in these studies provided contrasting results based upon their differential effects on β-arrestin2 signaling from the activated receptor. Herkinorin was utilized in these studies due to its distinctly different pharmacological properties when compared to morphine and DAMGO. Originally, herkinorin was identified as a structural derivative of salvinorin A [50], and a selective agonist for MOPr that does not recruit β-arrestin2 [18]. Herein we show that herkinorin does not induce β-arrestin2 recruitment to MOPr in primary sensory neurons, and produces antinociception without the development of OIH. Together with previous studies evaluating the development of tolerance [13], [48], these data further support the idea that MOPr agonists that do not effectively signal to β-arrestin2 hold promise as new therapeutics with reduced side effect profiles.

Several lines of evidence demonstrate the involvement of cross-talk in central mechanisms of OIH [51]–[53]. Although previous work indirectly postulates the contribution of a peripheral component to OIH [45], our findings directly evaluate the peripheral contribution of chronic MOPr activation to the development of OIH, and are the first to demonstrate β-arrestin2 cross-talk between MOPr and TRPV1. MOPr agonists such as morphine and DAMGO sequester β-arrestin2 to MOPr and thereby attenuate TRPV1/β-arrestin2 interactions to amplify TRPV1 activity in peripheral sensory neurons. This cross-talk mechanism is thought to contribute to symptoms of OIH that develop quickly following intraoperative administration of fentanyl or remifentanil [54], [55] or over time following methadone maintenance therapy [56]. Furthermore, patients discontinuing opioid therapy may experience rebound hyperalgesia due to overactive PKA, leading to phosphorylation and sensitization of TRPV1 [57]. Clinically, treatment options for OIH are extremely limited because increasing opioid administration exacerbates OIH and increases dependence [58].

We have previously shown that β-arrestin2 desensitizes TRPV1 in sensory neurons [8], so it is tempting to speculate that any chronic ligand treatment that could recruit β-arrestin2 away from TRPV1 in sensory neurons would lead to the development of OIH. Delta and kappa opioid receptor systems have also been shown to sensitize nociceptive processes, such as substance P and calcitonin gene-related peptide release [59], and lead to hyperalgesia *in vivo* [60], but studies have yet to evaluate the recruitment of β-arrestin2 to non-opioid receptor systems in the development of hyperalgesia or the potential clinical significance of hyperalgesia that may develop from chronic treatment with drugs that recruit β-arrestin2 to other receptor systems in peripheral sensory neurons. Therefore, mechanisms underlying OIH are complex and treatment options remain limited. Since herkinorin has poor blood-brain barrier penetrance [43], [61], and is capable of producing antinociception without sensitizing TRPV1 in peripheral sensory neurons, it might be a promising candidate for the treatment of pain. Peripherally-restricted MOPr agonists that do not recruit β-arrestin2 and thus selectively modulate the phosphorylation state of TRPV1 deserve consideration as potential treatments for inflammatory hyperalgesia, and potentially, to reduce OIH.
